# Transcriptome profiling of sheep granulosa cells and oocytes during early follicular development obtained by Laser Capture Microdissection

**DOI:** 10.1186/1471-2164-12-417

**Published:** 2011-08-18

**Authors:** Agnes Bonnet, Claudia Bevilacqua, Francis Benne, Loys Bodin, Corinne Cotinot, Laurence Liaubet, Magali Sancristobal, Julien Sarry, Elena Terenina, Patrice Martin, Gwenola Tosser-Klopp, Beatrice Mandon-Pepin

**Affiliations:** 1 INRA, UMR444 Génétique Cellulaire, Auzeville, BP52627, F-31326 Castanet-Tolosan, France; 2 INRA, UMR1313 Génétique Animale et Biologie Intégrative, Plateforme de Microgénomique expressionnelle ICE, F-78350 Jouy-en-Josas, France; 3 INRA, UR631 Station d'Amélioration Génétique des Animaux BP52627, F-31326 Castanet-Tolosan, France; 4 INRA, UMR1198 Biologie du Développement et de la Reproduction, F-78350 Jouy-en-Josas, France

## Abstract

**Background:**

Successful achievement of early folliculogenesis is crucial for female reproductive function. The process is finely regulated by cell-cell interactions and by the coordinated expression of genes in both the oocyte and in granulosa cells. Despite many studies, little is known about the cell-specific gene expression driving early folliculogenesis. The very small size of these follicles and the mixture of types of follicles within the developing ovary make the experimental study of isolated follicular components very difficult.

The recently developed laser capture microdissection (LCM) technique coupled with microarray experiments is a promising way to address the molecular profile of pure cell populations. However, one main challenge was to preserve the RNA quality during the isolation of single cells or groups of cells and also to obtain sufficient amounts of RNA.

Using a new LCM method, we describe here the separate expression profiles of oocytes and follicular cells during the first stages of sheep folliculogenesis.

**Results:**

We developed a new tissue fixation protocol ensuring efficient single cell capture and RNA integrity during the microdissection procedure. Enrichment in specific cell types was controlled by qRT-PCR analysis of known genes: six oocyte-specific genes (*SOHLH2*, *MAEL*, *MATER*, *VASA*, *GDF9*, *BMP15*) and three granulosa cell-specific genes (*KL*, *GATA4*, *AMH*).

A global gene expression profile for each follicular compartment during early developmental stages was identified here for the first time, using a bovine Affymetrix chip. Most notably, the granulosa cell dataset is unique to date. The comparison of oocyte vs. follicular cell transcriptomes revealed 1050 transcripts specific to the granulosa cell and 759 specific to the oocyte.

Functional analyses allowed the characterization of the three main cellular events involved in early folliculogenesis and confirmed the relevance and potential of LCM-derived RNA.

**Conclusions:**

The ovary is a complex mixture of different cell types. Distinct cell populations need therefore to be analyzed for a better understanding of their potential interactions. LCM and microarray analysis allowed us to identify novel gene expression patterns in follicular cells at different stages and in oocyte populations.

## Background

Many studies have been carried out to identify the mechanisms controlling early folliculogenesis (from follicle formation of the resting pool to the preantral stage). These early stages are important in regulating the size of the resting primordial follicle pool and the fate of the follicles, which in turn affects reproductive life span and fertility.

**Figure 1:**
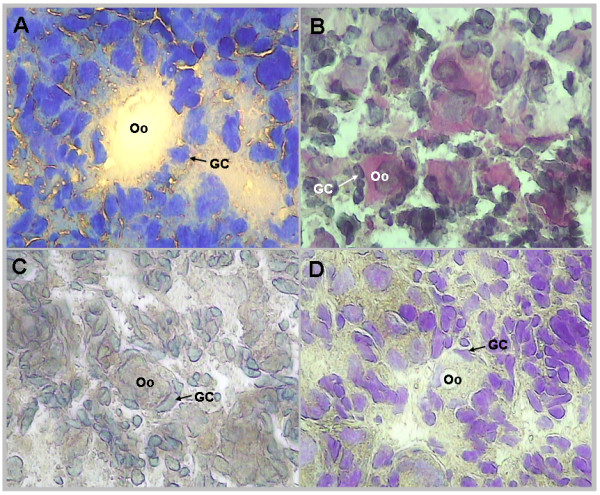
**Quality of tissue morphology with the four staining protocols**. New born ovary staining section (100 × magnifications) produced by: A. Toluidin blue. B. Hematoxylin Eosin. C. Histogen^®^. D. Cresyl Violet^®^. Abbreviations: Oo: oocyte, GC: granulosa cells.

Even if the events of folliculogenesis are well-conserved among mammals, differences exist between species in the timing of specific developmental changes and more specifically, the role of several genes was shown to be different between mono-ovulating and poly-ovulating species. The formation of primordial follicles occurs within a few days after birth in rodents and during fetal development in primates and ruminants such as sheep (75 days of gestation) [1]. The primordial follicles are composed of diplotene oocytes surrounded by flattened pregranulosa cells. Transcriptomic studies in human and in rodents, showed that a number of genes, such as transcription factors (Figure 1 alpha) [2], zona proteins, meiosis-specific enzymes and nerve growth factors [3] have already been identified as being involved in primordial follicle assembly. Once formed, primordial follicles remain in a dormant phase until they are recruited to initiate growth towards the primary stage. Orchestrated communication between oocytes and somatic cells (granulosa cells and thecal cells) is required during this transition and during the subsequent growth of follicles. Different components of the extra-cellular matrix, such as growth factors and cytokines, acting in an autocrine and paracrine manner, are involved in this cross talk. For example, cytokines such as the kit-ligand, expressed in granulosa cells, and its receptor c-kit, expressed in oocyte and theca cells [4], are involved in the formation of primary follicles. Then, oocyte-secreted growth factors such as GDF9 and BMP15 are involved in primary to secondary follicle transition [5,6]. They co-operate to regulate proliferation of granulosa cells [7]. In addition, other factors such as FOXL2, AMH or NGF also play a role in these different steps of early folliculogenesis [8].

Until now, efforts to discover genes have mainly focused on rodents and only a small number of genes are currently known, meaning that our basic understanding of the gene expression patterns driving early folliculogenesis is still very poor. More specifically, the expression of various oocyte-specific genes was demonstrated to be essential at a specific time during early folliculogenesis but little is known exactly about which granulosa cell factors play a specialized role in follicular development.

Moreover, all findings in rodent species are not necessarily applicable to other mammals, especially mono-ovulating species. Indeed, whereas mutations in the *BMP15 *gene cause infertility in ewes due to defects in folliculogenesis [9] and have been associated with premature ovarian failure (POF) in women [10-12], most defects in female mice lacking the bone morphogenetic protein BMP15 are confined to the ovulation process [13].

Finally, most transcriptomic studies of early folliculogenesis are based on homogenized tissues (whole ovary or follicles) [14-16], and do not take the multiplicity of cell types used in the analyses into consideration. Consequently, potentially valuable spatial information is lost and the minor cell components, expressed only in few cell types may be diluted below the level of detection.

Laser capture microdissection (LCM) allows precise microscopic isolation of pure cell populations from heterogeneous tissues for subsequent extraction and analysis of nucleic acids [17]. Conducting LCM experiments for gene expression profiling requires an acceptable tissue morphology to allow for histological selection of the desired cell type and to guarantee RNA integrity. Particularly, single cell LCM requires a lot of time to search for and identify cells of interest in the tissue section. Preserving RNA quality during this long process is challenging. Indeed, most existing protocols [18-22] do not guarantee the integrity of RNA during the long time required for microdissection.

In the present study, LCM was used to isolate oocytes and granulosa cells (GC) from new born sheep ovaries and their respective transcriptomes were compared using a 23 k bovine microarray and quantitative real-time PCR (qPCR).

We developed an LCM method allowing for a longer microdissection time without RNA degradation, to gain specific access to the follicular compartments, i.e. GC and oocytes, at each follicular developmental stage [23]. Starting from amplified RNA, gene expression profiles were monitored for each follicle population, according to cell type on an Affymetrix GeneChip Bovine array. Our results showed that microdissected samples were representative of the different compartments and stages and demonstrated the feasibility of using LCM and array hybridization to identify cell type and/or stage gene expression signatures in developing ovaries.

## Results

### LCM and RNA amplification

**Figure 2:**
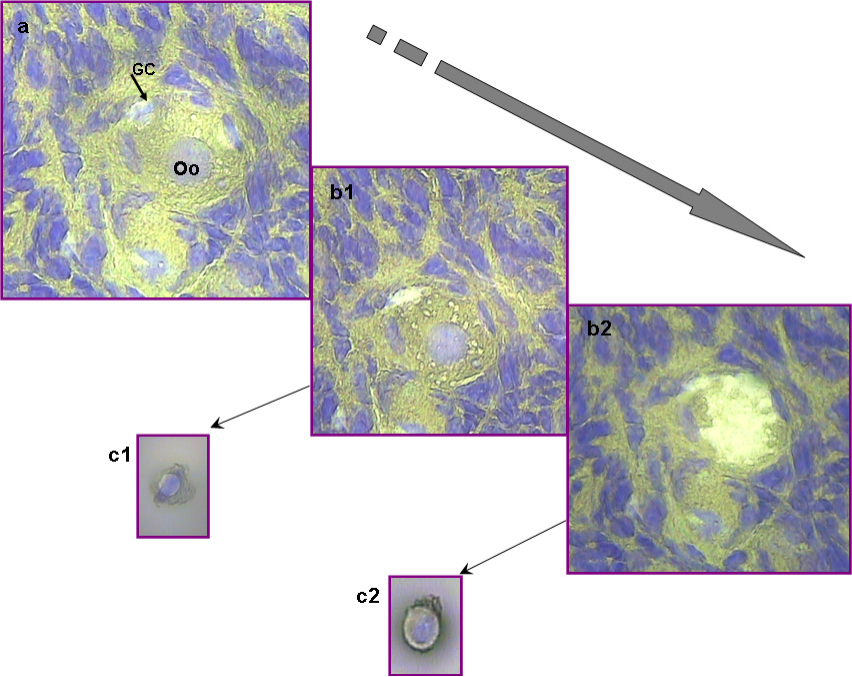
**Summary of the LCM capture of primordial follicular compartments**. Representative photographs of a primordial follicle before (a) and after (b) microdissection. GC were selected (a, arrow), dissected (b1) and collected in a cap (c1). The oocyte was dissected (b2) and collected (c2). Abbreviation: Oo: oocyte.

The purpose of any tissue preparation protocol for LCM is to obtain tissue sections allowing for unambiguous identification and successful capture of the cells of interest, while maintaining RNA integrity. Four staining protocols: Toluidin blue, Hematoxylin-Eosin (H&E), Histogen^® ^and Cresyl Violet^® ^were evaluated in terms of tissue morphology, maintenance of molecular integrity and capture success. Figure 1 shows that the best tissue morphology was provided by Cresyl Violet^® ^and H&E staining. The RNA Integrity Number (RIN) analysis revealed significant RNA degradation with Toluidin blue and Histogen^® ^staining (data not shown). Finally, although the Cresyl Violet^® ^protocol was the best both for identifying structures and for maintaining the best RNA quality, the ovary sections were fixed too tightly to the glass and capture did not exceed 50%. Consequently, we developed a new cold fixation protocol (70% ethanol fixation at -10°C), allowing efficient single-cell capture (Figure 2). In these conditions, RNA quality did not decrease more than 10% during the staining and microdissection period (up to 120 min). The RIN values typically obtained with RNA extracted from stained sections ranged between 6 and 8.7 and were similar to those obtained with standard fixation protocols (Additional file 1).

Using this protocol, the follicular compartments, i.e. GC and oocytes, were captured for each follicle stage: primordial (Pd), primary (Pm), secondary (Sec) follicles and the first antral stage as control (small antral: SA). After RNA extraction and amplification, the LCM-derived anti-sense-RNA (aRNA) samples appeared as a highly reproducible smear, ranging from 200 to 2000 bases in length across all samples (Additional file 2A). Table 1 summarizes the amount of estimated microdissected sections input and the aRNA yield for each LCM-sample.

**Table 1:** Microdissection and RNA yield

Follicular stage	Compartment	Replicates	Estimation of the number of sections input	aRNA yield (μg)
Primordial	Oocyte	3	130-160	7-28
	Granulosa	3	600	6-49
				
Primary	Oocyte	4	35-100	3-28
	Granulosa	4	100-490	2-24
				
Secondary	Oocyte	4	10-22	2-13
	Granulosa	4	Corresponding to 12-20 follicles	4-16
				
Small antral	Oocyte	2	13-15	4-20
	Granulosa	2	Corresponding to 12-13 follicles	44-55

Estimated number of microdissection sections input and corresponding yield of aRNA for each sample.

aRNA: amplified RNA

### Validation of sample specificity

**Figure 3:**
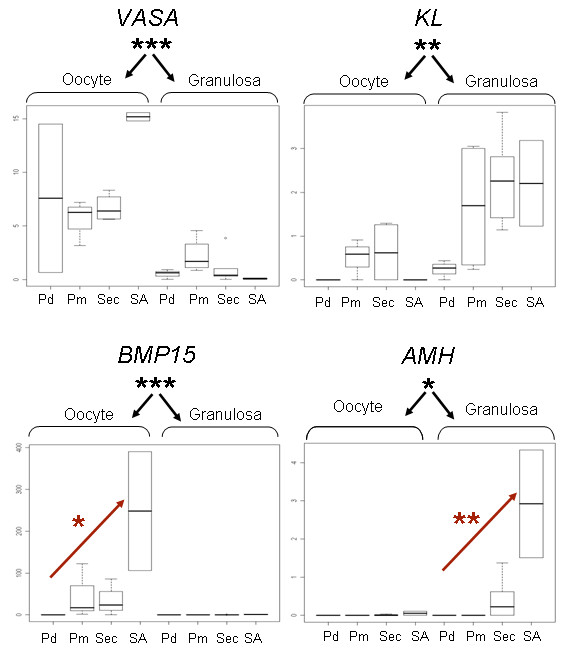
**Gene expression profiles**. Relative quantification throughout early follicular development and in the two follicular compartments (n = 3-4 except for Ant, n = 2). Profiles showed a significant increase in gene expression for *BMP15 *and *AMH *from the primary and secondary stage, respectively. By contrast, *KL *and *VASA *genes were expressed at all stages from the primordial follicular stage on, with no significant differential expression during the process of early folliculogenesis. The Y axis corresponds to the relative expression normalized by 2 reference genes (*Actin β *and *RPL19)*. The X axis corresponds to the different follicular stages of the 2 follicular compartments. Abbreviations: Pd: primordial, Pm: primary, Sec: secondary, SA: small antrum. *< 0.05, **< 0.01.

**Figure 4:**
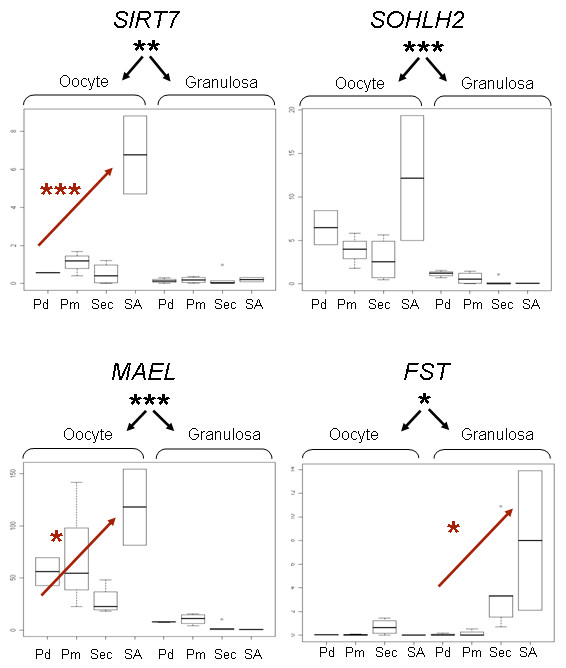
**SOLHL2, MAEL, *SIRT7 *and *FST *expression profiles**. Relative quantification of *SIRT7*, *SOLHL2*, *MAEL *and *FST *mRNA throughout early follicular development (Pd, Pm, Sec) and small antrum (SA) and in the 2 follicular compartments (n = 3-4 except for SA n = 2). The Y axis corresponds to the relative expression normalized by 2 reference genes (*Actin β *and *RPL19)*. The X axis corresponds to the different follicular stages of the 2 follicular compartments. Abbreviations: Pd: primordial, Pm: primary, Sec: secondary, SA: small antrum. *< 0.05, ***< 0.001.

The purity of the LCM-derived aRNA samples and the microarray data quality were examined by investigating specific gene expression of nine genes using quantitative real-time PCR (qPCR). Among them, six were oocyte-specific genes (*SOHLH2*, *MAEL*, *MATER*, *VASA*, *GDF9*, *BMP15*) and three known to be expressed in GC (*KL*, *GATA4*, *AMH*). Table 2 summarizes the results of the statistical analyses (see Material and Methods) for each gene. We identified significant interaction effects between compartments (oocyte, GC) and between stages (including early stages (Pd, Pm, Sec) and the first antral stage (SA)) for six genes. Significant differential expression between the compartments was confirmed for all the genes tested. Moreover, four genes showed a significant increase in expression associated with early follicular development (Table 2). For example, *BMP15*, *AMH *and *MAEL *expression increased between the primary and secondary developmental stage respectively. By contrast, *VASA, KL *and *SOHLH2 *displayed no significant differences between stages (Figure 3, 4). These results demonstrated that the LCM-derived aRNA samples were representative of the cell type and/or developmental stage concerned.

**Table 2:** Results of qPCR analysis

Gene	Microarray analysis			qPCR analysis			
		
		Compartment effect (O/GC)		Follicular stage		Interactions	
			
	O/GC Fold change	Fold change	P value class	Fold change	P value class	Compartment/stage	Expression profile Oocyte () GC ()
SOHLH2 VASA WEE	O only	194.5 184.6 2.2	*** *** *			*	
MAEL MATER GDF9 BMP15 SIRT7	2.3 1.5 1.3 5.2	229.4 14.3 374.5 316.7 33.0	*** * *** *** **	4.2 33.9 13.9 627.2 12.2	* *** * * ***	* *** ** * ***	
KL GATA 4 LAS1L PHGDH	-7.5 -2.1 GC only GC only	-3.6 -8.7 -1.8 -3.6	** * * NS: 0.067				
AMH FST	-7.3	-54.7 -697.5	* *	1608.8 122.3	** *	** *	

O/GC fold change corresponds to the highest oocyte expression of the 3 follicular stages versus the lowest ones. For genes overexpressed in GC, a minus sign was added to the GC/O fold change.

Follicular stage fold change corresponds to the highest expression stage versus the lowest within a compartment.

For each gene, a one-way ANOVA model was fitted with the 2 factors: "stage" (4 levels) and "compartment" (2 levels) with interactions using R statistical software.

The expression profile column visualizes the expression level of each compartment: bold line is used for oocyte expression and double line for GC expression. The slope symbolizes a significant increase of the expression during the follicular development.

P-value class: *: p < 0.05; **: p < 0.01; ***:p < 0.001; NS: non specific

Abbreviations: O: oocyte (red line), GC: granulosa cells (blue line)

### Global gene expression analysis

To explore global gene expression in the cell types and/or early developmental stages, the ovine LCM-derived aRNA samples were hybridized to the Affymetrix Bovine Genome Array, as generic microarray is not available in sheep, and the bovine and ovine genomes are phylogenetically close.

First, to assess the reliability of the heterologous hybridization on the bovine microarray, we compared hybridizations with RNA from bovine and ovine fetal gonads. Hybridization with the ovine fetal gonad RNA sample revealed an average of 49.8% of the probe sets called as "present" (scored probe sets detected using the Affymetrix algorithm) compared to an average of 57.1% for the bovine counterpart sample. Around 90% of these probe sets were shared (Additional file 3). The percentage of ovine transcripts detected in this control experiment is similar to a previous report using ovine mRNA on the Affymetrix GeneChip Bovine array [24]. This result demonstrated the possibility of using heterologous hybridization in our conditions.

Next, in order to keep LCM-derived aRNA samples for further expression experiments, we assessed the quality of Arcturus turbo™ labeling (indirect labeling after RNA amplification) compared to that of the Affymetrix labeling kit. Ovine fetal gonad RNA was subjected to three different labeling protocols (P1: Affymetrix standard target labeling, P2: two amplification rounds followed by Affymetrix cDNA labeling and P3: Arcturus turbo™ labeling kit) and hybridized to the Affymetrix Bovine Genome Array. The sensitivity and reliability of these protocols were evaluated using three measures of GeneChip performance (see Materials and Methods) provided by the Affymetrix algorithm (Additional file 3). The double round amplification data revealed a slight reduction in the number of "present" calls. The Scale Factor was similar in all hybridizations. Finally in labeling protocols from amplified RNA (P2; P3), the 3'/5' signal ratio was higher than the 3'/M ratio signal for two housekeeping genes (*GAPDH*, *GST*), demonstrating that the mean size of the aRNA obtained was shorter than Affymetrix standard labeling kit. Similar observations have been reported by others [25,26]. Since each round of amplification shortens the length of the RNA transcript, eventually resulting in the loss of 5' regions, the signal ratio between 3' and 5' probe sets consequently increases. In addition, the intensity signal of Arcturus turbo™ labeling aRNA showed a high level of concordance with the Affymetrix ones (r = 0.8).

The analysis of signal intensity values for the four *Bacillus subtilis *control transcripts that were added to all LCM-derived RNA samples before amplification (see Materials and Methods) showed comparable amplification in all the LCM-derived RNA samples and the ovine fetal ovary (r average: 0.92).

### Cell profiling

Different follicle population hybridizations enabled us to detect 11,291 probe sets corresponding to 8652 annotated transcripts, split into 6794 to 8344 probe sets according to the samples (Table 3).

**Table 3:** Microarray results

	Our data		Mouse data (pan, 2005) Unigene
		
	present probe sets	unique annotated genes	
PDO	7828	6300	9258
PMO	6794	5603	9494
SECO	7379	6039	9420
PDG	8344	6631	
PMG	6931	5708	
SECG	8092	6545	
Total detected	11291	8652	
Chip representation	24024	12404	25535

Distribution of the detected probe sets and unique annotated genes among the LCM-derived RNA samples.

'Total detected' refers to the whole detected probe sets within the LCM-derived RNA oocyte samples.

LCM-derived RNA oocyte samples were compared to Pan et al. data (Mouse oocyte data).

We also identified 1050 transcripts specifically expressed in GC and 759 transcripts expressed only in oocytes during early follicular development. Five genes (*KL*, *GATA4*, *MAEL*, *MATER*, *GDF9) *known as having compartment-specific expression were confirmed by microarray data and qPCR analyses (Table 2). Five additional genes (*SIRT7*, *FST, LASL1*, *WEE*, *PHGDH) *showing a new spatiotemporal expression were checked by qPCR and 4 of them were validated. Moreover, we observed a significant increase in *SIRT7, MAEL *and *FST *expression during early follicular development (Figure 4).

The biological relevance of the two specifically expressed gene lists (1050 + 759) was evaluated *in silico *using Ingenuity Pathway Analysis (IPA). A total of 1689 genes were identified as eligible by IPA and used to generate networks.

**Figure 5:**
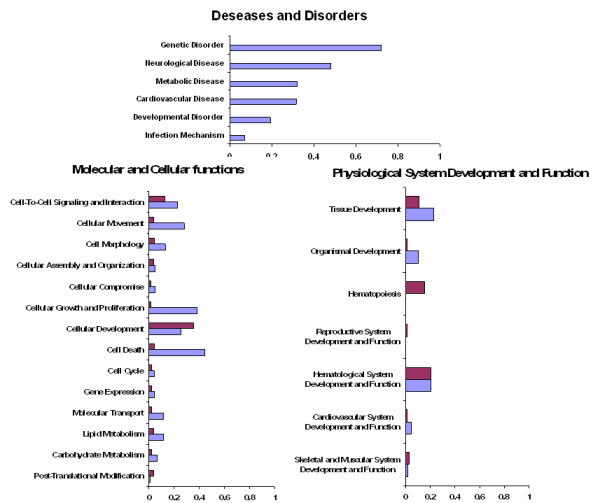
**Statistically significant enriched functions of oocyte and granulosa cells**. The 2 specifically expressed gene lists (oocyte/GC) were evaluated *in silico *using Ingenuity Pathway Analysis (IPA). Analysis revealed 3 statistically significant enriched categories (p-value < 10**^-3^**). The X axis corresponds to the ratio between the focus genes versus the genes of the class. GC data are in blue color and oocyte data are in red color.

Seven significant biological networks for oocytes and ten significant biological networks for GC were selected (score > 18, Additional file 4). These networks identified common and specific top functions (Figure 5) with their associated pathways (Additional file 5). The "Diseases and Disorders" catalog was specific to GC and included the most significant Genetic Disorder class, which contained 72% of the eligible genes. Genes from GC were also mainly involved in "Cell Growth and Proliferation", "Cell Death" and "Lipid Metabolism" classes. We also identified GC functions involved in "Cell Movement and Cell Interactions". The oocyte-specific gene list highlighted two specific functions: "Reproductive System and Function" that clearly targets folliculogenesis (including *GDF9, FOXOA1 *and *SYCP3 *genes) and the "Hematopoiesis Function" that mainly includes differentiation processes.

## Discussion

Gene expression profiling through the use of oligoarrays is a powerful approach for characterizing the whole population of transcripts in a tissue, and amplification methods now allow these techniques to be used for very rare material. Nevertheless, it is still difficult to obtain cell type specific expression profiles from heterogeneous tissues. For these reasons, in the context of early folliculogenesis, our current knowledge has been largely derived from rodent whole ovaries [14,27,28] and the few isolation protocols available referred to oocytes [29,30] or whole follicles [16,31] and did not differentiate GC. Although the expression profile of some compartment-specific genes could be inferred, the presence together of different cell types confused the analysis. Consequently, little information about the role of GC during early folliculogenesis is available.

The strength of the present study is to develop a method for obtaining reliable specific expression profiles and to characterize GC and oocytes separately at several key stages corresponding to major transitions during the development of the follicles.

### Quality of the LCM-derived aRNA

To obtain reliable microarray results, LCM must produce a sufficient amount of high quality RNA. In the case of single cell LCM, we have to face the long microdissection period required and the extraction of only a limited amount of RNA. In this study, our modified protocol allowed us to capture the cells and to preserve the RNA integrity of the two follicular compartments (Table 1) throughout microdissection (up to 120 min, see Additional Data 2). First, we showed that, as previously described in other tissues [32-35], histological and RNA quality of the staining are tissue specific (Figure 1) and probably depend on cell component reactions and intracellular RNAse content. The success of capture depends on a number of tissue-slide adhesion factors such as the type of slides used or the method and temperature of fixation [19]. Sluka *et al*. [35] found that maintaining testis tissue at a cold temperature during fixation and subsequent processing (e.g. staining) was important to maximize preservation of tissue morphology. As recently described in mammary gland [36], we showed in this study, that cold temperature was also important for the capture process.

Finally, our data showed that 500-600 single GCs were sufficient to produce enough aRNA to perform both microarray hybridization and qPCR experiments (Table 1). The decrease in the required number of oocytes captured according to the stage of follicular development is also in agreement with an increase in the diameter of the oocyte (34-73 μm) [23] and in their RNA content [37-39].

### Gene expression patterns

In this study, we documented nine ovine gene expression patterns in specific follicular compartments within *in vivo *microenvironment (Table 2). We confirmed cell type-specific expression in all the genes tested. As described by Luzzy *et al*. [40], we identified variability between the replicates (Figure 3) that could be associated with technical processing and/or with biological microheterogeneity and individual variability. The expression profiles of ovine *BMP15*, *GDF9 MATER*, *VASA *and *SOHLH2 *oocyte-specific genes and ovine *KL*, *GATA4*, *AMH *GC-specific genes are consistent with previous reports on sheep fetal ovaries [41], germinal vesicle (GV) oocytes [42] or other species [43-46]. In addition, we report here an increase in the expression of four cell type-specific genes during the early ovine folliculogenesis.

Our data provide additional clues about species specificities. They underline differences in *SOHLH2 *and *MAEL *gene expression patterns from those identified in rodent species (Figure 4). The *Sohlh2 *gene is a spermatogenesis- and oogenesis-specific transcription factor that was recently discovered in mouse species with a restricted pattern of expression in oocytes of primordial and primary ovarian follicles in immature ovaries [47-49]. By contrast with mouse species, we observed constant expression of *SOHLH2 *until the SA stage. Likewise, *mael *is a Drosophila spindle-class gene required for Drosophila oogenesis that localizes to nuage (nucleolus-like bodies) and is implicated in miRNA/RNAi pathways [50]. *Mael *is expressed in the early drosophila germ line [51]. In mouse, Gallardo *et al*. showed that expression of *Mael *is high in primordial oocytes but rapidly decreases during follicle growth to disappear in the SA follicles [14]. We revealed that the expression of *MAEL *is maintained until small antral follicles in sheep. Such *SOHLH2 *and *MAEL *expression patterns suggest the existence of different mechanisms as a function of species that will need further investigation. This underlines the importance of acquiring expression data from different species and highlights certain species specificities.

### Accuracy of array data

The originality of this preliminary study was to identify global gene expression in oocyte and GC separately for key stages of early folliculogenesis. Several observations support the accuracy of the expression data.

First, the level of "present" call probes from the oocyte samples (43%) and the size of the annotated transcripts detected (Table 3) were in accordance with mouse oocyte data (43-47% of "present" call probes) [30] and bovine oocyte data (54% of "present" call probes) [52].

**Figure 6:**
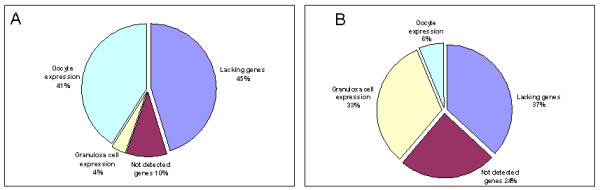
**Accuracy of the gene data sets**. Comparison of A - oocyte data sets and B - GC data sets with Arraztoa [29], Dadé [53], Gallardo [14] and Pan [30] data sets.

In addition, we also identified the great majority of genes previously found to be expressed in the four following studies: Dadé *et al*. [53], Arraztoa *et al*. [29], Pan *et al*. [30] and Gallardo *et al*. [14] (Additional file 6). The results of these studies mainly derived from oocytes (mouse and monkey species). Figure 6A shows that 41% of the oocyte genes reported in these studies were expressed in our sheep oocyte samples. Our data on GC during early folliculogenesis are quite unique, as even if some information about GC expression was previously available [14,30], it only concerned very few genes (33 genes). Figure 6B shows that 33% of these genes were also detected in our GC data sets. These percentages take into account the percentage of undetected genes (10 to 24%: Figure 6) which could be partly due to the relative specificity of heterologous hybridizations and the high percentage of missing genes (37 to 45%: genes reported in the previous studies that were missing in the bovine Affymetrix chip used here). This underlines the fact that genomics of livestock animals have to face with incomplete arrays and/or incomplete genome annotations [54].

### Biological significance

To confirm that these data are biologically meaningful and a rich source for functional analysis, two lists of compartment-specific genes were generated and subjected to Ingenuity Pathway Analysis (IPA).

This functional analysis identified the three main cellular events known to be involved in early follicle development (Figure 5) [23]. First, we observed three cellular functions in GC ("Cell Movement", "Cellular Assembly and Organization", and "Cell Morphology") probably associated with the first cellular event, the switch of cell shape from flattened to cuboidal [55]. Second, the identification of genes involved in "Cellular Growth and Proliferation" and "Cell Death" is correlated with the second cellular event: the marked increase in the number of GC (× 40 in ovine species up to the secondary stage) [23], a key process in the initiation and the development of primordial follicles. Finally, in oocytes, the "Axonal Guidance Signaling" pathway (Additional file 5) and the "Cellular Development" function are consistent with the third cellular event: oocyte enlargement. Axonal guidance signaling refers to the different receptors and signal transduction cascades that drive axons towards their synaptic targets and finally lead to the reorganization of the cytoskeleton and confer the adhesive properties the cell requires for its spatial organization. This pathway was previously mentioned in the analysis of regulatory gene networks involved in primordial follicle development [56].

In addition, the importance of the crosstalk between the oocyte and GC during follicular development [57,58] may be illustrated by the expression of genes of the BMP signaling pathway in GC in response to oocyte BMP15 production (Additional file 7). This crosstalk was also highlighted by the "Cell to Cell Signaling and Interaction" function in both GC and oocytes (Figure 5) and "Inflammatory and Immune Response" mechanisms found in GC. These mechanisms featured the "Cytokine Signaling" pathway, "Cellular Immune Response" pathways and the "Genetic Disorder" class (genes mainly linked to inflammatory response mechanisms of the colon). They include members of the *TGFβ *gene family such as AMH [46], and cytokines encoding genes such as KL [4] both expressed in GC and known to interact with oocytes and to play an essential role in early folliculogenesis. We also identified the specific GC expression of different interleukins such as the proinflammatory cytokine IL1-β previously reported in antral follicles for their involvement in ovarian steroidogenesis, in the ovulation process and in oocyte maturation [59,60] and which are up-regulated during primordial-primary follicle transition (from rat ovary culture experiments) [61]. Interestingly, the IL1 receptor gene (*IL1R*) appears to be only expressed in oocytes, which supports the hypothesis of cytokine oocyte-GC crosstalk. However, the exact function of these genes in this context remains to be elucidated.

Thanks to these specific gene lists, we were able to show for the first time that expression of the *SIRT7 *gene is confined to the oocyte in sheep follicles (Figure 4). This gene was found to be expressed in most mouse and human tissues including -at a lower level of expression- the ovary [62,63]. The detailed expression pattern of the current study completes a previous rat expression pattern comparing ovaries enriched in different follicle populations [61]. The *SIRT7 *gene belongs to the sirtuin family and is involved in the regulation of a wide range of biological processes such as gene silencing, aging, cellular differentiation, and metabolism [64]. In human, the colocalization of SIRT7 with Polymerase I and the nucleolar transcription activator UBF suggest that SIRT7 is a positive regulator of Pol I transcription [65]. Its oocyte expression is consistent with the high demand for protein synthesis for oocyte growth. Like the *basonuclin *gene [66], this expression suggests a role for SIRT7 in the transcriptional regulation of sheep oocyte rDNA that will need further experiments to verify nuclear localization. Finally, this study underlines the precise expression pattern in GC of the *FST *gene that plays an important role in female fertility by regulating activin and members of the BMP subfamily [67]. Different studies have shown induction of the *FST *gene expression by recombinant human BMP15 [68] and suggested an inhibitory action of FST on BMP15 function [69]. Its strongly increased expression at the secondary stage in sheep (Figure 4) combined with the *BMP15 *oocyte-expression pattern corroborate the results of these studies and are consistent with the BMP15 function to enable the primary-secondary transition.

## Conclusions

In the present study, LCM and microarray analysis were used as tools to identify distinct gene expression patterns in ovary cell populations and led to the identification of genes of potential significance in female fertility. The data obtained here are in agreement with the literature regarding oocyte and GC-specific transcript lists. We identified specific expression patterns in sheep (*SOHLH2, MAEL, SIRT7*, *FST*). Our findings show that the mRNA repertoire in the different cell types is regulated dynamically during the transition from primordial to intermediate follicles.

This study is a starting point for further investigation of the GC compartment in particular. Specific gene expression patterns in the oocyte and GC could be of great interest for deciphering the critical molecular processes and the complexity of the communication between these two compartments, who enable the folliculogenesis process to occur as it is expected.

## Methods

### Tissue processing and staining

This study was conducted in compliance with institutional guidelines for research studies (animal experimentation authorization no 31-297, prefecture de la Haute Garonne). Twelve lambs from Langlade INRA experimental farm (Castanet-Tolosan, France) were euthanized at birth (< 1 day) with 3 ml of Dolethal (Vetoquinol, ALCYON ZI, France). The ovaries were removed and embedded in O.C.T. embedding matrix (CML, ref KMA-0100-00A, France), frozen in liquid nitrogen and stored at -80°C until use.

Eight-micrometer serial frozen sections were cut on a cryostat at -20°C, mounted on cooled (4°C) sterile microscope glass slides and stored in a 50 ml plastic tube inside the cryostat chamber (-15°C) for few minutes [36]. The sections were fixed 30 s with 70-75% ethanol (Fluka, Ref: 02855, Sigma-Aldrich) and stained individually at room temperature before LCM. Four different staining protocols were compared: Toluidin blue, Hematoxylin-Eosin (H&E), Histogene^® ^staining solution (Arcturus, Applied Biosystems^®^) and Cresyl Violet^®^, LCM staining kit Ambion, Applied Biosystems^®^). The sections were conserved under vacuum until LCM capture (< 1 day). The experiment was performed in triplicates.

#### Hematoxylin and eosin staining

water15 s, Hematoxylin solution 5 s (Sigma-Aldrich, Ref: MHS16), Scott water 5 s (Sigma-Aldrich, ref: S5134), water 2 s, 75% ethanol 30 s; 95% ethanol 30 s, diluted eosin (1/4) 5 s (Sigma-Aldrich, ref: HT110316), 75% ethanol 30 s, 95% ethanol 30 s, (100% ethanol 30 s) × 2, (xylene 5 mn) × 2 (Sigma-Aldrich ref: 296325).

#### HistoGene™ LCM staining

(Arcturus (Applied Biosystems^®^), ref: KIT0415) was performed according to the manufacturer's recommendations.

#### Toluidin blue staining

Toluidin blue in alcoholic solution 1 mn (Sigma-Aldrich ref: 31393-1G), water 15 s, (95% ethanol 30 s) × 2, (100% ethanol 1 mn) × 2, (xylene 5 mn) × 2.

#### Cresyl violet staining

(Ambion Europe Ltd, LCM Staining Kit Ref AM 1935): 50% ethanol 20 s, cresyl violet 20-30 s, 50% ethanol 20 s, 75% ethanol 30 s, (95% ethanol 40 s) × 2, (100% ethanol 1 mn) × 2, (xylene 5 mn) × 2.

Staining protocols were evaluated in terms of tissue morphology, capture success and maintenance of molecular integrity. The influence of the staining method on RNA quality was assessed by controlling the quality of total RNA extracted from tissue scrapes on the slide as described in the RNA extraction section, prior to and after fixation/staining steps using an Agilent 2100 bioanalyzer.

The capture was improved by taking slide temperature, fixation temperature and fixation duration into account. The best capture efficiency for each section was obtained by using a cooled slide (4°C) that was fixed 30 sec with a ice cold 70% ethanol solution (-20°C).

### Laser capture microdissection

The follicular compartments (*i.e*. granulosa cells (GC)) and oocytes were selected for each follicle stage (primordial (Pd), primary (Pm) and secondary (Sec) follicles and small antral (SA) as control) under microscope taking the observation of section series into account and according to the classification by Lundy *et al *[23]. Briefly, the following criteria for the follicle stage selection were applied: i) primordial follicles (Pd) were defined as an oocyte surrounded by 2-3 of flattened GC and no cuboidal GC, ii) primary follicles (Pm) were defined by a monolayer of cuboidal GC whatever the oocyte size, iii) secondary follicles (SEC) were defined by two layers of GC and iv) small antral follicles (SA) had a diameter less than 500 μm diameter. Dissections were carried out under 40× magnification microscopic visualization using the VERITAS™ and ARCTURUS XT apparatus (Arcturus, Applied Biosystems^®^). For the capture, the laser pulse was carefully adjusted for each section, so as to touch only the oocyte or the GC (laser length: 40-60 mw, duration: 10-20 ms). Taking care to not collect possibly atretic follicles (pycnotic oocytes or fragmented nuclei, shredded ooplasm or disintegrated follicular structures), we were able to identify and collect into separate CapSure™ HS LCM caps (Arcturus, Ref LCM 0214) oocytes and GC for the different follicular stages. After two hours of microdissection, the cap was removed, treated with 10-15 μl of extraction buffer from Picopure RNA Isolation kit (Arcturus, ref: KIT0202) and stored at -80°C until use.

### RNA extraction

Total RNA from pooled section scrapes corresponding to each cap was prepared by pipeting 50 μL of extraction buffer (Picopure RNA Isolation kit) directly onto the tissue section on the glass slide, using the pipette tip to gently scrape the tissue into the buffer Samples were then transferred into an RNase-free microcentrifuge tube and stored at -80°C. Total RNA was extracted using the PicoPure RNA Isolation Kit according to the manufacturer's protocol, including on-column DNase treatment (Qiagen, ref 79254, Courtaboeuf, France). The quality of the RNA was assessed using an Agilent 2100 bioanalyzer (Agilent Technologies, Palo Alto, CA) with an RNA6000 Pico Lab Chip and analyzed by the RNA Integrity Number (RIN) algorithm [70].

For LCM-derived samples, as only very few cells were captured per cap, the caps were pooled before total RNA extraction, according to the quality of the total RNA extracted from their corresponding sections scrapes. Finally, each LCM-derived RNA sample (pool of 2 to 20 caps coming from 1 to 4 animals) was performed in triplicate/quadruplets.

### T7 linear amplification

To generate sufficient RNA quantities for Genome-wide microarray analysis, LCM-derived RNA samples were subjected to 2 rounds of T7 linear amplification: aRNA was generated using RiboAmp^® ^HS PLUS kit (Arcturus, ref KIT0525), following the manufacturer's protocol. This protocol specified that a minimum input of 100-500 picograms of total RNA are required for successful amplification with this kit designed specifically for low-input total RNA samples, which is equivalent to 10-50 cells. The RiboAmp HS process has been already validated for generating highly reproducible microarray data, and the gene expression profiles obtained using this method have shown high fidelity when compared with profiles generated from unamplified samples [40]. After in vitro transcription, the optical density of antisense RNA (aRNA) was measured at 260 and 280 nm. The yield and the size distribution of each aRNA sample were evaluated using the Agilent Bioanalyzer 2100 with a RNA 6000 Nano Lab Chip. In complement, four *B*. *Subtilis *control transcripts (Affymetrix, GeneChip Eukaryotic Poly-A RNA Control Kit, ref 900433) were added to each LCM-derived RNA sample before amplification. Based on the average of 500 pg of starting RNA (about 50 whole cells) the following serial dilutions were used: 1:20, 1:50, 1:50, 1:10, 1:20 and 1 μl of the final dilution 1:5 was added per RNA sample.

### Microarray experiments

Ovine microarray experiments were performed using the Affymetrix Bovine Expression Array (representing approximately 23,000 transcripts). The quality of the cross-species hybridizations was checked by comparison of hybridization data of ovine fetal ovary RNA with bovine fetal ovary RNA, generated with the standard protocol (one Affymetrix round amplification).

Three biotin-labeling protocols were compared from ovine fetal ovary total RNA:

- Protocol 1: 3 μg of total RNA were labeled according to the standard Affymetrix protocol (one round of amplification [71,72]).

- Protocol 2: total RNA was subject to two-rounds of amplification using the RiboAmp^® ^HS PLUS kit until the second-round cDNA synthesis. Biotin-labeled cRNAs were synthesized following the Affymetrix protocol using the second-round cDNAs as templates (440 ng/8 μl).

- Protocol 3: total RNA was subject to two round of amplification using the the RiboAmp^® ^HS PLUS kit. Fifteen micrograms of aRNA were labeled using the Arcturus biotin turbo™ labeling kit (Arcturus, ref KIT0608) [34].

Finally, one LCM-derived aRNA sample from each condition coming from pools of different animals (Primordial, Secondary stages: 2 animals and Primary stage: 4 animals) was labeled using the Arcturus biotin turbo™ labeling kit (protocol 3).

In accordance with the Affymetrix technical manual, 10 μg of cRNA was purified, fragmented and hybridized at the concentration of 40 ng cRNA/μl hybridization mix to Affymetrix Bovine Expression Arrays, in the Microarray Core Facility of the Institute of Research of Biotherapy, CHRU-INSERM-UM1 Montpellier.

### Data management

All microarray CEL files from this study comply with MIAME standards. They have been deposited in GEO (http://www.ncbi.nlm.nih.gov/geo), accession number GSE25652, using the BASE software adapted by SIGENAE bioinformatics platform (http://www.sigenae.org).

### Microarray data analysis

Signal intensities and detection call of probe sets from each hybridized genechip were extracted using the Affymetrix software microarray suite 5 (MAS 5), using the default parameters.

In LCM-derived aRNA sample hybridizations, all probe sets called "absent" on all chips in the image interpretation were filtered out. Because the experimental design did not include technical or biological replicates we applied a stringent filter to the probe sets detected. For the transcripts, with different probe sets called as "present", we selected the most in 3' UTR of the gene and taking into account the probe set repetitions. Indeed, for half of the genes, the Affymetrix GeneChip system contains more than one probe sets, providing a high number of repeated experiments within a single chip for hybridization validation. However, the discordant probe set signals within a transcript may be the result of the ovine on bovine heterologous hybridization. Finally, only transcripts with a signal in all their probe set replicates were considered as ''detected".

The integrity of the RNA and the quality of hybridizations were evaluated using three measures of GeneChip performance provided by the Affymetrix algorithm: the number of "present" calls, the Scale Factor and and the 3'/5' signal ratio. The number of "present" calls and the Scale Factor (multiplier used to normalize the whole chip to a target intensity of 100; inversely related to chip brightness) are sensitive to RNA sampling, labeling, scanning and data extraction. The 3'/5' signal ratio is designed to detect the 3' and 5' regions of the mRNA; the 3'/middle ratio refers to the mRNA transcript from 3' to the middle of the mRNA transcript. These values provide information about the quality and the integrity of the RNA.

### Probe set annotations and biological network analysis

Primary annotation of probe set sequences were obtained from the NetAffx Analysis Center. Probe set sequences were also compared with Human and Bos Taurus refseq_rna NCBI databases by BLAST to confirm and complete Affymetrix annotations (performed by Sigenae team). Transcripts were then discussed by gene name (HUGO gene symbols).

Ingenuity^® ^Pathway Analysis software (IPA; http://www.ingenuity.com) was used to examine biological functions and molecular pathways. This software combines functional annotations of our selected genes (focus genes) and the corresponding bibliographic data to generate significant canonical pathways and biological networks.

Biological analysis was focused on the two lists of genes specifically expressed in oocytes or in GC. The Bovine Genome Array was used as set reference. A threshold network score of 18 corresponding to the highest score obtained with the gene set reference was applied to select the highest significant networks for further analysis. Then, these selected networks were explored to identify statistically significant functional categories (p-value < 10^-3^) and canonical pathways (p-value < 5 10^-2^).

### Quantitative RT-PCR

Gene primer designs were performed from ovine mRNA, ovine EST or ISGC Data sequences (https://isgcdata.agresearch.co.nz/). Gene primers were designed in the 3' UTR in the first 1000 base pairs using LightCycler Probe Design2 software (Roche Diagnostics). The intron-exon organization of ovine genes was deduced by comparison with the Human genome using the Iccare software [73]. The primer pairs were confirmed by Primer3 (http://frodo.wi.mit.edu/primer3/). Sequences are available in Additional file 8.

The LCM-derived aRNA samples were reverse transcribed in a reaction volume of 20 μL combining 500 nanogram of aRNA, 250 nanogram of random hexamers (Promega, ref: C1181, Charbonnieres, France), 200 mM dNTP, 10 mM DTT, 4 μl of 5× SuperScript II First-Strand Buffer, 40 U RNase inhibitor (Promega, Ref: N2111), and 200 U of SuperScript II (Invitrogen, ref: 18064-014, Cergy Pontoise, France). The reaction was incubated according to the manufacturer's protocol. The reactions were completed to 50 μl and diluted at 1/12.5 before PCR. The assay for each gene consisted of 4-5 replicates per condition (except for SA = 2) and negative controls.

The ovine fetal ovary total RNA sample was reverse transcribed using Primer p(dT)15 for cDNA synthesis (Roche Diagnostic, ref 10814270001, Meylan, France) according to the manufacturer's protocol.

QPCR was performed from 3 μl of the final dilution using SYBR green fluorescence detection during amplification on an ABI Prism 7900 Sequence Detection System 2.1 (Applied Biosystems^®^) as previously described [74]. The real-time PCR amplification efficiency was calculated for each primer pair using six serial dilution points from the ovine fetal ovary cDNA sample (1:9; 1:3; 1:3; 1:3; 1:2; 1:2). After determination of the threshold cycle (Ct) for each LCM-derived aRNA sample, the PFAFFL method was applied to calculate the relative expression of each gene [75] using the 1:120 ovine fetal ovary cDNA dilution as calibrator sample.

The relative expression was normalized by the corresponding geometric average of two reference genes using geNorm v3.4 [76]: *β-actin *gene that was slightly expressed in our micro-array experiment and *RPL19 *gene that was found to be highly expressed and not regulated during follicle development.

The significance of the relative expression data was tested using the one-way ANOVA models of R statistical software system (the Comprehensive R Archive National, http://www.cran.r-project.org). Models on raw data outlined a clear violation of the hypothesis of variance homogeneity. Therefore data were log transformed. No departure from model hypotheses was found in that case. For each gene, an ANOVA model was fitted, with the 2 factors "stage" (4 levels) and "compartment" (2 levels) with interactions. A backward variable selection procedure was applied for each gene, to decide whether the 2 factors have significant effects on the expression of the considered gene, that is: interaction effects, additive effects, "compartment" effect only, "stage" effect only or not differentially expressed. For that purpose, F tests were applied (lm and ANOVA functions in R).

## Authors' contributions

AB conceived and designed the study, performed LCM experiments, analyzed the microarray data, carried out the RT-PCR and statistical analyses, and was responsible for much of the writing. FB and JS participated in obtaining embedded ovary and staining sections. MS, helped in the statistical analyses. ET, CB and PM contributed to the development of the LCM protocol. LB provided the animals. LB, CB, CC, LL, PM, MS, GTK and BMP contributed to the conception and design of the study, and were involved in the drafting and revising the manuscript. All authors read and approved the final version of the manuscript.

## Supplementary Material

Additional file 1 **RNA integrity along LCM**. Influence of the fixation step and microdissection time on RNA quality. Y axis: the quality of total RNA extracted from each staining section was checked using an Agilent 2100 bioanalyzer. X axis: time required for microdissection of each section. The pink line corresponds to RIN from a staining section fixed with 70% ethanol. The blue line corresponds to RIN from a staining section fixed with 75% ethanol. Click here for file

Additional file 2 **Quality control of labeled aRNA integrity**. One microliter of labeled or unlabeled primordial granulosa cell aRNA was analyzed on an RNA LabChip and Agilent Bioanalyser. **A**: unlabeled aRNA. **B**: aRNA labeled with the Turbo labeling Kit (biotin). Click here for file

Additional file 3 **Microarray performance**. Comparison of methods used to generate biotinylated cRNA (see material and methods) using quality control measures: % "present call" probe sets is the percentage of scored probe sets detected by the Affymetrix Microarray suite 5.0 (MAS.5.0) Scale Factor is the multiplier used to adjust the trimmed mean signal of a probe array to a selected target signal value (100 by default). 3'/5' ratio is the ratio of the 3' probe set signal intensity to the 5' probe set signal intensity of a transcript. 3'/M ratio is the ratio of the 3' probe set signal intensity to the Medium (M) probe set signal intensity of a transcript. Click here for file

Additional file 4 **Functional networks for compartment-specifically expressed genes (oocyte and GC)**. Click here for file

Additional file 5 **Canonical pathways of ocyte and granulosa cells**. Ten statistically significant enriched canonical pathway categories (p-value < 5 10**^-2^**) were revealed in the specific oocyte and granulosa cell gene lists. The X axis corresponds to the ratio of focus genes to pathway genes. Red and blue numbers correspond to the number of focus genes that contributed to the pathway (oocyte vs GC). Click here for file

Additional file 6 **Comparison of gene data sets with the literature**. Literature data: 1 - Dadé: Differentially expressed genes in mouse oocytes compared to other tissues. The selection was performed by *in silico *differential display between 3 mouse oocyte cDNA libraries and 13 selected tissues cDNA libraries. 2 - Gallardo: set of ovarian factor from mouse Foxo3 ovaries. Gene classes were revealed by comparative profiling from Mouse RNA affymetrix hybridization data sets including ovary RNA extracted at four time points spanning follicle assembly and early growth, and 14 somatic tissues containing LCM primary oocytes and LCM somatic cells. 3 - Pan: Mouse oocyte differentially genes expressed between primordial and primary follicular stages. Overall change in oocyte gene expression was characterized using Pd, Pm, Sec, SA and antral mouse follicles. 4 - Arraztoa: Primate oocyte-enriched transcripts between microdissected primordial stage and placenta RNA (control). Click here for file

Additional file 7 **BMP signaling pathway: an example of compartment crosstalk**. From the microarray data, we visualize the focus genes involved in the BMP signaling pathway. The genes specifically expressed in the oocytes are in red color and the genes specifically expressed in GC are in green color. Click here for file

Additional file 8 **Primer sequence (5'3') for real-time PCR**. Click here for file
