# Supplementary figures and images for: Transcriptome profiling of sheep granulosa cells and oocytes during early follicular development obtained by Laser Capture Microdissection
Source: BMC Genomics. 2011 Aug 18;12:417. doi: 10.1186/1471-2164-12-417 (PMC3166951; doi:10.1186/1471-2164-12-417)

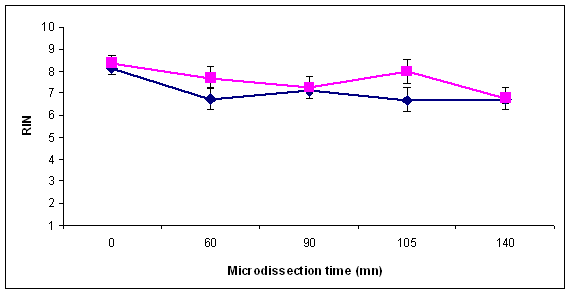

Supplement: Additional file 1 — RNA integrity along LCM. Influence of the fixation step and microdissection time on RNA quality. Y axis: the quality of total RNA extracted from each staining section was checked using an Agilent 2100 bioanalyzer. X axis: time required for microdissection of each section. The pink line corresponds to RIN from a staining section fixed with 70% ethanol. The blue line corresponds to RIN from a staining section fixed with 75% ethanol. [file 1471-2164-12-417-S1.TIFF]

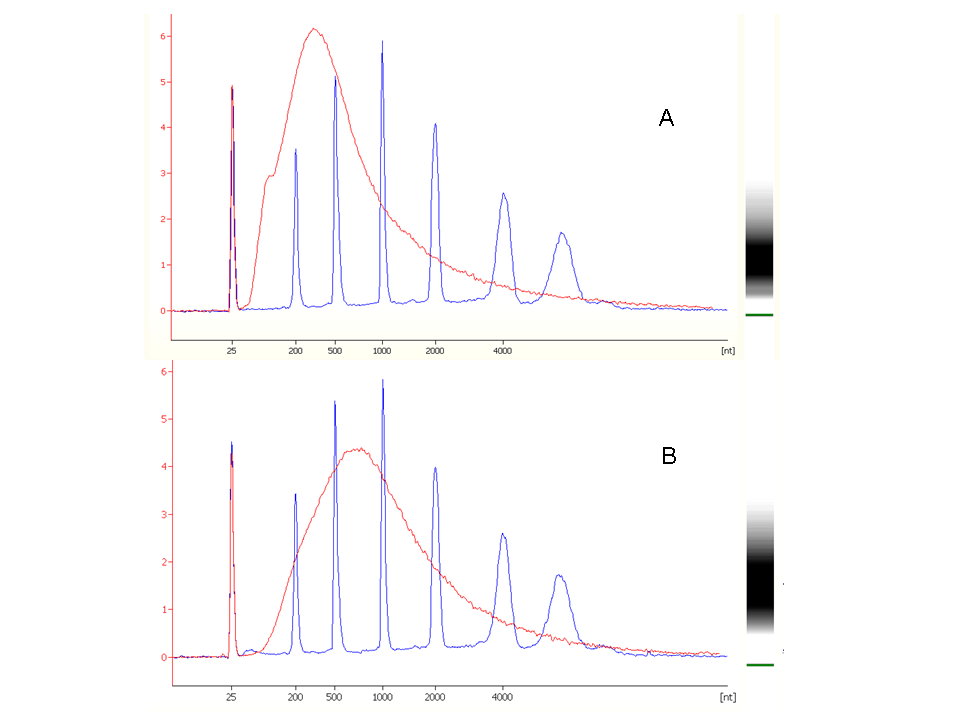

Supplement: Additional file 2 — Quality control of labeled aRNA integrity. One microliter of labeled or unlabeled primordial granulosa cell aRNA was analyzed on an RNA LabChip and Agilent Bioanalyser. A: unlabeled aRNA. B: aRNA labeled with the Turbo labeling Kit (biotin). [file 1471-2164-12-417-S2.TIFF]

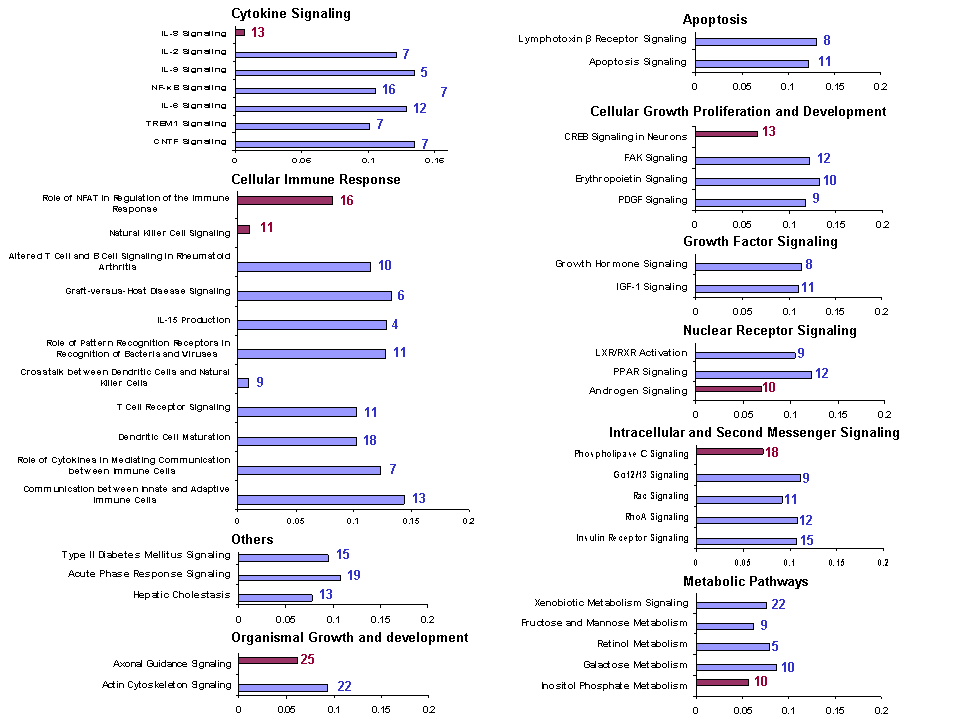

Supplement: Additional file 5 — Canonical pathways of ocyte and granulosa cells. Ten statistically significant enriched canonical pathway categories (p-value < 5 10-2) were revealed in the specific oocyte and granulosa cell gene lists. The X axis corresponds to the ratio of focus genes to pathway genes. Red and blue numbers correspond to the number of focus genes that contributed to the pathway (oocyte vs GC). [file 1471-2164-12-417-S5.TIFF]

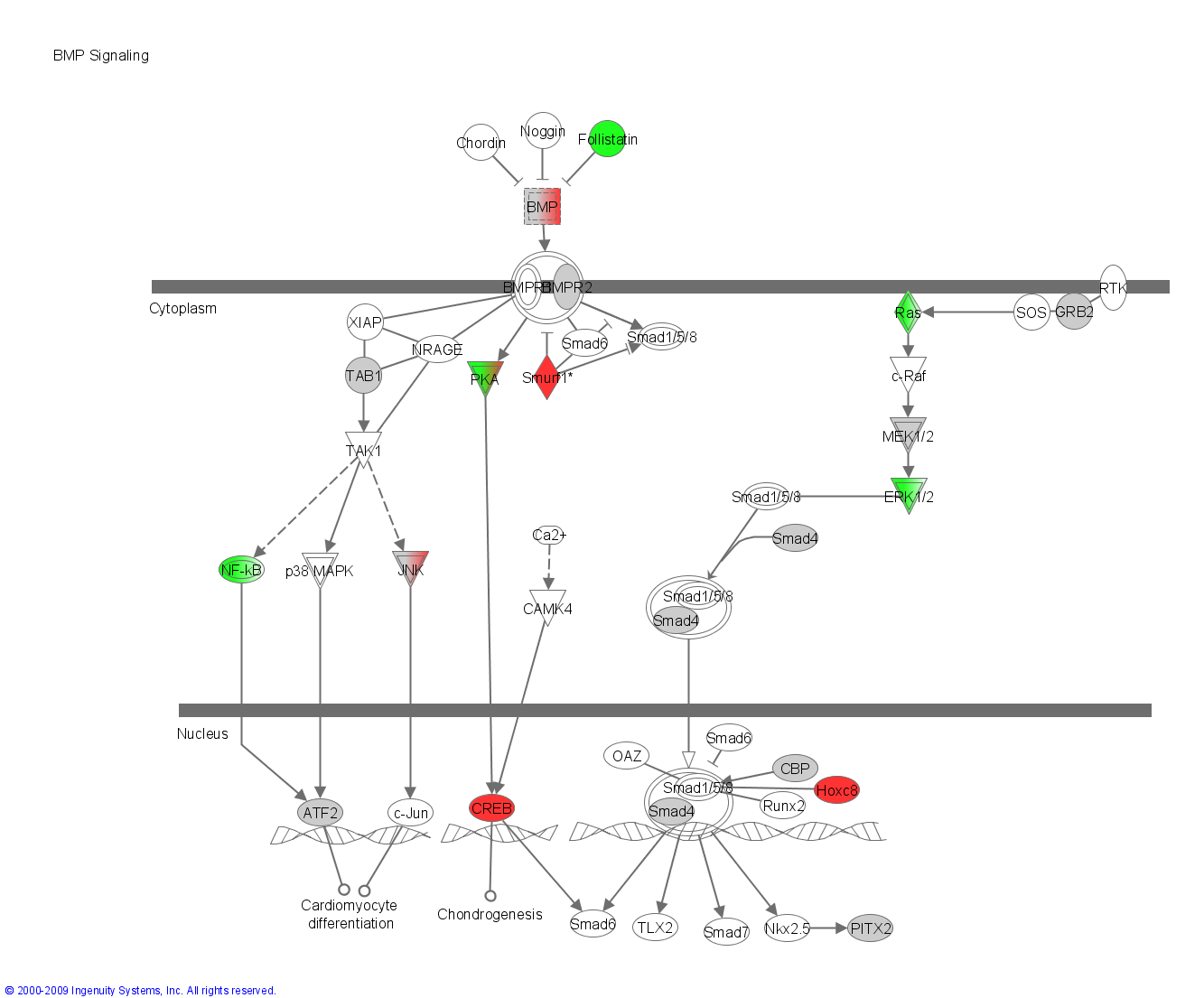

Supplement: Additional file 7 — BMP signaling pathway: an example of compartment crosstalk. From the microarray data, we visualize the focus genes involved in the BMP signaling pathway. The genes specifically expressed in the oocytes are in red color and the genes specifically expressed in GC are in green color. [file 1471-2164-12-417-S7.TIFF]
