# Supplementary material for: Transcriptome profiling of sheep granulosa cells and oocytes during early follicular development obtained by Laser Capture Microdissection
Source: BMC Genomics. 2011 Aug 18;12:417. doi: 10.1186/1471-2164-12-417 (PMC3166951; doi:10.1186/1471-2164-12-417)
Supplement: Additional file 3 — Microarray performance. Comparison of methods used to generate biotinylated cRNA (see material and methods) using quality control measures: % "present call" probe sets is the percentage of scored probe sets detected by the Affymetrix Microarray suite 5.0 (MAS.5.0) Scale Factor is the multiplier used to adjust the trimmed mean signal of a probe array to a selected target signal value (100 by default). 3'/5' ratio is the ratio of the 3' probe set signal intensity to the 5' probe set signal intensity of a transcript. 3'/M ratio is the ratio of the 3' probe set signal intensity to the Medium (M) probe set signal intensity of a transcript. [file 1471-2164-12-417-S3.DOC]

| Samples | Protocol | % Hybridized Probe sets "present call" | Scale factor | GAPDH 3'/5' ratio | GAPDH 3'/M ratio | GST 3'/5' ratio | GST 3'/M ratio |
| --- | --- | --- | --- | --- | --- | --- | --- |
| Bovine gonad | Protocol 1 | 57.1 | 3.79 | 1.17 | 1.13 | 0.53 | 0.96 |
| Ovine gonad | Protocol 1 | 49.8 | 3.42 | 1.19 | 0.8 | 0.08 | 0.5 |
| Ovine gonad | Protocol 2 | 38.6 | 5.1 | 67.67 | 10.76 | 1.49 | 2.86 |
| Ovine gonad | Protocol 3 | 37.8 | 6.53 | 112.08 | 14.98 | 1.14 | 1.54 |
| PDO | Protocol 3 | 34.13 | 3.00 | 20.82 | 13.84 | 2.54 | 4.45 |
| PMO | Protocol 3 | 29.39 | 4.87 | 19.66 | 19.84 | 1.08 | 2.03 |
| SECO | Protocol 3 | 31.96 | 4.59 | Na | 25.19 | 2.23 | Na |
| PDG | Protocol 3 | 36.52 | 2.36 | 10.58 | 13.89 | 0.5 | 1.42 |
| PMG | Protocol 3 | 30.12 | 4.83 | 31.25 | 21.08 | 4.5 | 5.03 |
| SECG | Protocol 3 | 35.41 | 3.03 | 23.85 | 18.55 | 1.57 | 2.68 |
