# Supplementary material for: Transcriptome profiling of sheep granulosa cells and oocytes during early follicular development obtained by Laser Capture Microdissection
Source: BMC Genomics. 2011 Aug 18;12:417. doi: 10.1186/1471-2164-12-417 (PMC3166951; doi:10.1186/1471-2164-12-417)
Supplement: Additional file 4 — Functional networks for compartment-specifically expressed genes (oocyte and GC). [file 1471-2164-12-417-S4.DOC]

| ID | Analysis | Molecules in Network | Score | Focus Molecules | Top Functions |
| --- | --- | --- | --- | --- | --- |
| 1 | Granulosa | ADORA2B, ADRA1B, ALB, AMBP, APOA1, BMP1, C4ORF41, CCDC88C, CCL3, CCR1, CHEMOKINE, CHRM4, CLCA2 (includes EG:9635), CXCL10, DNTT, FST, Gpcr, GPLD1, IGHG1, IRF3, ITIH5, KRT7, LGMN, MAP7D1, P2RY4, PDZRN4, peptidase, Pld, PLD4, PROC, SCN5A, STX1B, STXBP1, SYT7, TPP1 | 41 | 31 | Infection Mechanism, Cardiovascular Disease, Tachycardia |
| 2 | Granulosa | ACTA1, Actin, ANXA13, AP4M1, CNN1, CSN1S1 (includes EG:1446), DCTN6, DNAJC13, DVL1, FCER1A, Filamin, FLNB, GPR37, HECW1, Hsp70, Ige, INPPL1, IPP, KLHL2, LALBA, MYRIP, NCALD, OSBP, PACRG, Pak, PAK6, PFN1, PLEC1, PPM1E, Profilin, ROCK1, SMG5, TFRC, VIPR1, XPO6 | 38 | 29 | Tissue Development, Cellular Assembly and Organization, Cellular Compromise |
| 3 | Granulosa | BCAS3, BCL9, C21ORF33, Cbp/p300, CD8A, CHRNA3, CISH, Creb, CREB5, Cyclin A, DAB2IP, DNMT3B, E4F1, EPO, ERAF, G6PD, HIRIP3, Histone h3, Histone h4, HMGA2, IL15, MPZ, MRAS, NCF1, NDUFA3, NDUFB5, OPN1LW (includes EG:5956), PER2, Ras, RASSF1, RIN1, SATB1, SHOC2, SMC4, TBL1X | 38 | 29 | Cell Cycle, Cell-To-Cell Signaling and Interaction, Cellular Growth and Proliferation |
| 4 | Granulosa | ADIPOQ, AQP7, CCL22, CD80, CD3EAP, CIITA, DGAT1, DNA-directed RNA polymerase, HSPA1A, IKBKAP, IL1, IL8, IL-1R, IL1A, IL1B, IL1RAP, NFKB2, NFkB (complex), NFKBIA, PDE8A, PDZK1, PMM1, POLR1A, POLR2L (includes EG:5441), POLR3K, PPARG, PRMT2, Pro-inflammatory Cytokine, RELB, RNA polymerase II, SWI-SNF, TAF1A, TCERG1, TNNT1, ZBTB7A | 36 | 28 | Gene Expression, Lipid Metabolism, Molecular Transport |
| 5 | Granulosa | ACIN1, ADAM2, ADAM8, ADAM17, Akt, ATPase, CARD11, CD53, CSK, DCK, DDX19B, DLG1, ERCC3, FZD4, GRB10, GRID2, INSR, Integrin, KITLG, MAD2L1, Metalloprotease, MIR1, MYLK2, NP, Phosphorylase, PREX1, RUVBL2, SHANK2, SYT17, TAOK3, Transferase, TSC1, TSC22D4, TSPAN4, UBD | 36 | 28 | Cardiac Output, Cardiovascular System Development and Function, Cell Cycle |
| 6 | Granulosa | ABL1, AKAP13, ATN1, ATXN7, BAG1, BCAR3, ENOX1, Estrogen Receptor, Gcn5l, GIT1, GM2A, HEXA, ITGB3BP, LIFR, LSS, LYZ, MAX, N-cor, NEDD9, NR1H3, OSMR, PIP4K2A, POU1F1, PPARGC1B, Proteasome, PSMB10, Rb, RCC1 (includes EG:1104), Rxr, SETD7, STAM, TAF6, TOM1, tyrosine kinase, Ubiquitin | 34 | 27 | Genetic Disorder, Metabolic Disease, Neurological Disease |
| 7 | Granulosa | C1q, CD34, CD36, CD93, COL18A1, Collagen type I, Collagen(s), Complement component 1, CTSS, ERP44, FCGBP, FLI1, FMOD, GLYCAM1, HAPLN1, HSD11B1, IgG, IL2, IL18, IL12 (complex), IL12B, ITGB6, NCAN, PRRX1, PTX3, SELP, SERPING1, SRGN, Tgf beta, TGM2, TH1 CYTOKINE, Thyroid hormone receptor, TNC, TNFAIP6, ZNF384 | 32 | 26 | Cell-To-Cell Signaling and Interaction, Tissue Development, Carbohydrate Metabolism |
| 8 | Granulosa | 14-3-3, Alcohol group acceptor phosphotransferase, ATYPICAL PROTEIN KINASE C, Calmodulin, CDK5, DKC1, DUSP26, EIF2AK2, ERCC6L, ERK, G6PC, GRK4, Hsp90, HSPB6, Ikk (family), IQGAP1, IRAK1, KIF3A, KSR1, LLGL1, LYPLA2, MAP2K2, MAP2K5, MAP3K6, MAPK3, Mek, NEFM, Par6, PARD6B, PIM1, PLK1, PRKAA1, PRKCI, Rac, RGN | 30 | 25 | Amino Acid Metabolism, Post-Translational Modification, Small Molecule Biochemistry |
| 9 | Granulosa | ATP6V0C, CES5, CES2 (includes EG:8824), ELMO1, ELMOD2, HADHB, HDAC8, HNF4A, HPX, IFRD2, KIF3B, MIR124-1, MLKL, NUAK1, OSBP, PARP4, PARP16, PBX2, PGM1, PIGS, PNPLA6, PODXL, PROP1, RHOG, ROCK1, S100A9, SLC17A5, SLC39A7, SSFA2, STIM1, SULT1A1, TBC1D15, TCIRG1, TLE3, USP2 | 21 | 20 | Carbohydrate Metabolism, Cell Death, Developmental Disorder |
| 10 | Granulosa | C3ORF34, C8ORF41, CCDC82, CEBPB, DLG4, E2F4, GUF1, HNF4A, ISOC1, L2HGDH, MRTO4, MSRB2, NUDT11, ONECUT1, ORMDL1, SEPX1, SGSH, SH3BGRL2, TMEM176A, TRAF6, TRMT6, VHL, ZNF146 | 19 | 16 | Post-Translational Modification, Gene Expression, Cellular Development |
| 1 | Oocyte | 14-3-3, AFP, Akt, AKT1S1, BAG2, BMP15, C5ORF22, CA9, CTNNAL1, DCC, DYRK1B, E2f, E2F1, ELOF1, FOXA1, GDF9, Histone h3, HN1, IL1R1, MLXIP, MTUS1, MYB (includes EG:4602), NEK1, NFKB1, NFKBIL2, NR5A2, P38 MAPK, PI3, PRLR, RHOBTB2, SKA2, THAP7, TRIB3, TSC2, ZNF350 | 45 | 30 | Cell-To-Cell Signaling and Interaction, Reproductive System Development and Function, Tissue Development |
| 2 | Oocyte | Ap1, APOB, ARG1, ATF3, BATF, CAMP, CEBPG, CNKSR1, Creb, CREB1, Cyclin A, DBT, DNER, DUSP10, EIF2S2, FANCC, FEN1, GIPC2, GTF2H3, Hsp70, Jnk, MAFK, MAPK10, NFkB (complex), QTRT1, Ras, Ras homolog, RHOG, RHOT2, RHPN1, RNASEH2A, RPA2, SEMA4B, TNFRSF4, UNC119 | 38 | 27 | Organismal Development, Gene Expression, Cell Morphology |
| 3 | Oocyte | Actin, Alpha tubulin, ATPase, CNP, COTL1, CRLF3 (includes EG:51379), DMAP1, DNASE1, DPYSL2, EPN2, F Actin, FHIT, GDA, MIR124, MYH1, MYH8, MYH9, Myosin, OCLN, Pak, PAK4, PFDN4, PHLDA1, PTBP1, PXN, Rac, RASGRP2, SLC15A4, SNRPA, STMN4, TBCD, Tubulin, VPS4B, WASF2, WBP4 | 36 | 26 | Cellular Movement, Nervous System Development and Function, Cellular Assembly and Organization |
| 4 | Oocyte | Calmodulin, CENPE, CENPF, CPNE2, CSNK1G2, CYP26A1, CYP4F2, DIO1, DRD5, DSP, EID1, Estrogen Receptor, GNA12, HEY1, Histone h4, Hsp90, MTA1, PCNT, Phosphoinositide phospholipase C, PLC, PLCB3, PLCD1, PLCZ1, PPP5C, Rar, Rb, RUNX2, Rxr, RYR1 (includes EG:6261), SLC9A1, SNAPC3, SOST, TRIM24, UBAC1, UBR4 | 35 | 26 | Cell Morphology, Skeletal and Muscular System Development and Function, Cell-To-Cell Signaling and Interaction |
| 5 | Oocyte | ACTN3, Alpha Actinin, CD37, CD82, COL10A1, Collagen type I, Collagen(s), CSPG4, FGB, Fibrinogen, ICAM1, IGF2, IGFBP5, Integrin, Integrin alpha 3 beta 1, Integrin alpha 4 beta 1, Integrinα, ITGA3, ITGB2, LAMA3, Laminin, LGALS3, LMO7, LTBR, MHC Class II, Mmp, MMP13, MMP19, MMP23B, MYPN, PDLIM2, PLG, SERPINC1, XPNPEP2, ZYX | 30 | 24 | Cell-To-Cell Signaling and Interaction, Tissue Development, Cellular Movement |
| 6 | Oocyte | ACP1, BCR, BLNK, CD3, CD8, CD38, CD79B, CD8B, CUL4A, DDB2, IFN ALPHA RECEPTOR, Igm, KLRA1 (includes EG:10748), LAT, LCK, MED15, MHC Class I (complex), MHC CLASS I (family), NCK, PRUNE, PTPN6, PTPN22, RBBP5, RNA polymerase II, SLA2 (includes EG:84174), SLC27A2, SLC9A8, Sos, SYK/ZAP, TCR, TNNT2, TRIM11, VAV, XAB2, ZAP70 | 26 | 22 | Cellular Development, Hematological System Development and Function, Hematopoiesis |
| 7 | Oocyte | ATG9A, BAT4, C6ORF165, C6ORF170, CCDC49, CCDC85B, CEBPZ, CHCHD3, ETV6, ETV7, FAM164C, FAM50B (includes EG:26240), FBXL6, FBXL7, FBXO32, HNF4A, HNRNPC, IPO13, KDM2B, KIAA1045, L3MBTL, MIR293, NFYB, PLXNC1, PRRG2, RBM41, SFRS17A, SKP1, SLC25A28, SLC39A8, STAT4, TLE6, TM7SF3, ZNF524, ZNF764 | 20 | 18 | Gene Expression, Cell Cycle, Cellular Movement |
