# Supplementary material for: Transcriptome profiling of sheep granulosa cells and oocytes during early follicular development obtained by Laser Capture Microdissection
Source: BMC Genomics. 2011 Aug 18;12:417. doi: 10.1186/1471-2164-12-417 (PMC3166951; doi:10.1186/1471-2164-12-417)
Supplement: Additional file 6 — Comparison of gene data sets with the literature. Literature data: 1 - Dadé: Differentially expressed genes in mouse oocytes compared to other tissues. The selection was performed by in silico differential display between 3 mouse oocyte cDNA libraries and 13 selected tissues cDNA libraries. 2 - Gallardo: set of ovarian factor from mouse Foxo3 ovaries. Gene classes were revealed by comparative profiling from Mouse RNA affymetrix hybridization data sets including ovary RNA extracted at four time points spanning follicle assembly and early growth, and 14 somatic tissues containing LCM primary oocytes and LCM somatic cells. 3 - Pan: Mouse oocyte differentially genes expressed between primordial and primary follicular stages. Overall change in oocyte gene expression was characterized using Pd, Pm, Sec, SA and antral mouse follicles. 4 - Arraztoa: Primate oocyte-enriched transcripts between microdissected primordial stage and placenta RNA (control). [file 1471-2164-12-417-S6.DOC]

| **References** | **Species** | **Compartment** | **Data** | **Identified gene number** | **Bovine Affymetrix chip** | **Our data set** | | |
| --- | --- | --- | --- | --- | --- | --- | --- | --- |
|  |  |  |  |  | **Present gene number** | **Detected gene number** | **Oocyte detected gene number** | **Granulosa detected gene number** |
| **Dadé (1)** | Mouse | oocyte | enriched by In silico DD | 104 | 69 | 60 | 57 |  |
| **Gallardo (2)** | Mouse | oocyte | Class IA-follicle assembly/meiosis | 32 | 8 | 6 | 6 |  |
|  |  |  | Class IC-oocyte-specific, early maturation only | 14 | 3 | 3 | 3 |  |
|  |  |  | Class IB-oocyte-specific, early, and late maturation | 66 | 12 | 9 | 7 |  |
|  |  |  | Class III-Unfertized egg | 84 | 50 | 37 | 31 |  |
|  |  | somatic cells | Class ID-follicle growth, somatic | 24 | 12 | 8 |  | 6 |
| **Pan (3)** | Mouse | oocyte PD/PM | increase | 197 | 116 | 89 | 80 |  |
|  |  |  | decrease | 213 | 135 | 120 | 109 |  |
|  |  |  | somatic cells | 9 | 9 | 5 |  | 5 |
| **Arraztoa (4)** | Monkey | oocyte PD | enriched/placenta | 79 | 44 | 36 | 31 |  |
