# Supplementary material for: Transcriptome profiling of sheep granulosa cells and oocytes during early follicular development obtained by Laser Capture Microdissection
Source: BMC Genomics. 2011 Aug 18;12:417. doi: 10.1186/1471-2164-12-417 (PMC3166951; doi:10.1186/1471-2164-12-417)
Supplement: Additional file 8 — Primer sequence (5'3') for real-time PCR. [file 1471-2164-12-417-S8.DOC]

| gene | Reference | Up primer | Down primer |
| --- | --- | --- | --- |
| GDF9 | [NM_001142888.1](http://www.ncbi.nlm.nih.gov/entrez/viewer.fcgi?val=NM_001142888.1) | CAACACTGTTCGGCTCTTCA | CAGCAGATCCACTGATGGAA |
| BMP15 | AF236078S2 | CATGATGGGCCTGAAAGTAAC | CCCGAGGACATACTCCCTTA |
| MATER | AY721594 | GCTGGAGGCGTGTGGACTG | GGTCTGTAGATTAGAGGTGGGATGC |
| MAEL | CU653136 | CAAACACACCCACTGGTGAC | GTATCCGTGTTTTGGGGATG |
| SOHLH2 | CU638088 | ACAGCACACCCCAACTGTCT | GGCACCCAATAGGTGGTCTA |
| VASA | AF541971 | CGAGGGCTGGATATTGAAAA | TGCCAGTATTTCCACCACGA |
| SIRT7 | EE831540 | TGCAGCTCCTCATGGAT | ACTATGGCTGCCTTCTT |
| AMH | EV97COZ06DJDPJ* | CCTCAGTCGGACCGCAA | TTGCCTGTGTAGGCTGT |
| FST | M63123 | TCCAGGCAGCTCTACAT | GGGTAGGTCACTCCATCA |
| GATA 4 | XM_616466.4 | CCCGGAGGTAGCAGAGTTAT | TGTGGGTTAGGGAAGGGTAT |
| KITLG | EWZ7IHI02DESHV* | CACAGGATAACTTTGAGTGC | TGTGTATGTGTATGTACTGTAAGTGT |
| ACTINE β | NM_001009784 | CCAGCACGATGAAGATCAAG | ACATCTGCTGGAAGGTGGAC |
| RPL19 | AY158223 | CACAAGCTGAAGGCAGACAA | TGATGATTTCCTCCTTCTTGG |

* available at <https://isgcdata.agresearch.co.nz/>
